# Raptor regulates functional maturation of murine beta cells

**DOI:** 10.1038/ncomms15755

**Published:** 2017-06-09

**Authors:** Qicheng Ni, Yanyun Gu, Yun Xie, Qinglei Yin, Hongli Zhang, Aifang Nie, Wenyi Li, Yanqiu Wang, Guang Ning, Weiqing Wang, Qidi Wang

**Affiliations:** 1 Shanghai National Clinical Research Center for Endocrine and Metabolic Diseases, Key Laboratory for Endocrine and Metabolic Diseases of Chinese Health Ministry, Ruijin Hospital Affiliated to Shanghai Jiaotong University School of Medicine, 200025 Shanghai, China; 2 Sino-French Research Center for Life Sciences and Genomics, Ruijin Hospital Affiliated to Shanghai Jiaotong University School of Medicine, 200025 Shanghai, China

## Abstract

Diabetes is associated with beta cell mass loss and islet dysfunctions. mTORC1 regulates beta cell survival, proliferation and function in physiological and pathological conditions, such as pregnancy and pancreatectomy. Here we show that deletion of *Raptor*, which is an essential component of mTORC1, in insulin-expressing cells promotes hypoinsulinemia and glucose intolerance. *Raptor*-deficient beta cells display reduced glucose responsiveness and exhibit a glucose metabolic profile resembling fetal beta cells. Knockout islets have decreased expression of key factors of functional maturation and upregulation of neonatal markers and beta cell disallowed genes, resulting in loss of functional maturity. Mechanistically, *Raptor*-deficient beta cells show reduced expression of DNA-methyltransferase 3a and altered patterns of DNA methylation at loci that are involved in the repression of disallowed genes. The present findings highlight a novel role of mTORC1 as a core mechanism governing postnatal beta cell maturation and physiologic beta cell mass during adulthood.

## Abstract (web summary)

mTORC1 regulates beta cell survival, function and adaptation to physiologic and pathological stimuli. Here Ni *et al*. demonstrate that that deficiency of Raptor, a component of mTORC1 complex, impairs insulin secretion and glucose tolerance in mice by affecting maturation of beta cells during the postnatal period.

Type 2 diabetes is characterized by hyperglycaemia due to insulin resistance in peripheral tissues and a defect of insulin secretion resulting from continuous loss of functional beta cell mass^1^. In the adult pancreas, functional beta cell mass is not only determined by the number of beta cells, but also by their functional maturation status, including the ability to produce and secrete mature insulin in response to elevated blood glucose level^2^,^3^. Dedifferentiation of mature beta cell to a progenitor-like state under metabolic stress is one of the mechanisms of beta cell failure in type 2 diabetes^4^. Several transcription factors (*Pdx1, Nkx6.1, NeuroD1, MafA, Islet-1*)^5^,^6^,^7^,^8^,^9^, cellular signals (thyroid hormone, CTGF)^10^,^11^ and nuclear receptor ERRγ (ref. 12) have been reported to be crucial for maintaining beta cell identity and functional maturation. Yet, it remains unclear which signalling pathway determines beta cell functional maturation and mass expansion after birth. Fetal beta cells are poorly responsive to glucose^13^, but undergo a dramatic process of functional maturation in the early postnatal period, between day 2–8 (ref. 14), characterized by acquisition of the ability to secrete insulin in response to glucose^15^.

mTOR signalling is composed of a number of highly conserved Ser/Thr protein kinases, functioning in the form of at least two large protein complexes, mTOR complex 1 (mTORC1) and mTOR complex 2 (mTORC2)^16^. mTORC1 consists of RAPTOR (regulatory associated protein of mTOR), mLST8, PRAS40, DEPTOR and mTOR, which is sensitive to rapamycin^17^. mTORC1 pathway is recognized as a key factor to integrate signals from nutrients, growth factors and hormones, and controls cellular processes, including protein synthesis, ribosome biogenesis and lipogenesis by modulating eukaryotic translation initiation factor 4E binding protein 1 (4E-BP1) and ribosomal protein S6 kinase 1 (S6K1) (ref. 18). Multiple studies have clarified the role of mTORC1 signalling in beta cell function and mass. First, *in vitro* studies using rapamycin demonstrated that mTORC1 regulates beta cell function and survival in beta cell lines and murine/human islets^19^. Second, we and others have reported that rapamycin *in vivo* prevented beta cell adaptation to challenges, such as pregnancy^20^ and 60% partial-pancreatectomy^21^. Third, stimulation of mTORC1 activity in beta cells by overexpression of *Rheb* (ref. 22) or disruption of *Tsc1*/*Tsc2* (refs 23, 24) increased beta cell mass and improved glucose tolerance. Fourth, global *S6k1* deficient and *Rps6* knockout mice exhibited hypoinsulinemia and glucose intolerance, with diminished beta cell size^25^,^26^,^27^. Observations from the genetic models targeting component of the mTORC1 pathway suggest that mTORC1 is a key signal to regulate beta cell mass; nevertheless, its effect on beta cell proliferation and apoptosis remains controversial. Recent work using conditional *Raptor* knockout mice demonstrated tissue-specific mTORC1 functions in controlling whole-body metabolism^28^,^29^,^30^. Currently, the role of *Raptor* in beta cells remains unknown.

In the present study, we use beta cell specific *Raptor* knockout mice and report a direct link between mTORC1 signalling and beta cell functional maturation, which is an important and novel field of beta cell research. There exist multiple layers of regulation, including protein/insulin synthesis, translational capacity, cell size, mitochondria metabolism and DNA methylation. *Raptor*-induced beta cell functional immaturity precedes the onset of hyperglycaemia, which leads to beta cell failure.

## Results

### Impaired glucose tolerance and insulin secretion in βRapKO mice

**Figure 1:**
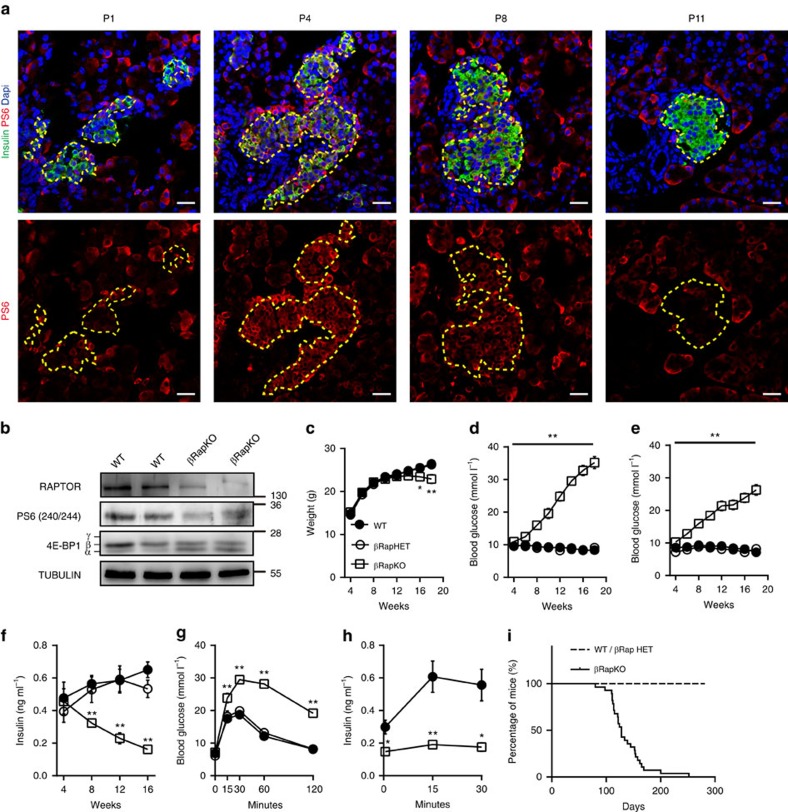
Deletion of *Raptor* in adult beta cells results in hyperglycaemia. (**a**) Representative pancreatic sections from WT mice at P1, P4, P8 and P11 were immunostained for PS6 (red) and insulin (green) (*n*=3). Scale bars, 20 μm. (**b**) Western blot showed decreased mTORC1 downstream targets phosphorylation of PS6 and 4E-BP1 in islets of 8-week-old βRapKO mice (*n*=3). (**c**–**e**) Body weight (**c**), random blood glucose (**d**) and 6-h fasting blood glucose (**e**) were monitored biweekly in WT (filled circles, *n*=14), βRapHET (empty circles, *n*=10) and βRapKO (empty squares, *n*=21) mice. (**f**) Six hours fasting insulin level from at least six mice of each genotype were measured at age of 4, 8, 12, 16 weeks. (**g**) Intraperitoneal glucose tolerance tests were performed on 8-week-old WT (*n*=12), βRapHET (*n*=7) and βRapKO (*n*=9) mice. (**h**) Insulin levels were determined after a glucose stimulus in 8-week-old WT and βRapKO mice (*n*=6). (**i**) Survival curve of WT, βRapHET and βRapKO mice (dashed lines: WT, *n*=14 and βRapHET, *n*=10; Solid lines: βRapKO, *n*=21). Results were presented as mean±s.e.m. of independent experiment indicated as above, *P<0.05, **P<0.01, unpaired Student's *t* test for two groups or ANOVA for multiple groups.

In preliminary studies, we examined the expression of mTORC1 target PS6 in pancreas sections during the transition from early neonatal life to post-weaning (Fig. 1a). IHC analysis of pancreatic sections showed that PS6 protein could be detected in some insulin^+^ cells from P1 pups, but strongly increased in nearly all insulin^+^ cells between P4 and P8 (Fig. 1a). By P11 just after the end of the maturation window, fewer beta cells showed staining for PS6 (Fig. 1a). Thus, expression of PS6 coincides with the transition from ‘immature' to ‘mature' glucose-stimulated insulin secretion (GSIS) response between 2 and 8 postnatal days (ref. 14), raising the possibility that the mTORC1 pathway plays a role in beta cell functional maturation.

To investigate the role of mTORC1 in mature beta cells, we generated mice lacking the key mTORC1 component *Raptor* specifically in beta cells (βRapKO). Successful knockout of *Raptor* was confirmed by western blot: RAPTOR was selectively absent in islets from 8-week-old βRapKO mice (Supplementary Fig. 1a). In addition, the mutant islets showed reduced phosphorylation of mTORC1 targets 4E-BP1 and PS6 (Ser240/244) (Fig. 1b). 4E-BP1 dephosphorylation was reflected in the shift from the highly phosphorylated γ-band to the nonphosphorylated α-band and an intermediate β-band (Fig. 1b). Thus, βRapKO mice are specifically defective in mTORC1 signalling in beta cells.

*Raptor* heterozygous mutant mice (βRapHET) exhibited similar weight, blood glucose levels, plasma insulin concentrations and survival rates as their littermate controls carrying the floxed allele of *Raptor* (WT) (Fig. 1c–g,i). βRapKO mice were born in the expected Mendelian ratio and did not differ in body weight from WT (Fig. 1c). However, the mutant mice started to display elevated random-fed glucose and 6-h fasted glucose level at the age of 4 weeks, and their glycemic control worsened with age (Fig. 1d,e). This rise was associated with significantly lower 6-h fasted plasma insulin levels in mutant animals, as early as 8 weeks after birth (Fig. 1f). We next measured blood glucose and plasma insulin levels after intraperitoneal glucose injection in 8-week-old βRapKO and WT: there was no significant difference in fasting glucose concentration, but a dramatic increase in glycaemia was observed in βRapKO mice following glucose challenge (Fig. 1g). As expected, these mutant mice exhibited lower basal insulin concentrations and mounted a poor insulin response when challenged with glucose (Fig. 1h). βRapKO mice showed a significant decrease in body weight at the age of 16 weeks compared with their age-matched littermates (Fig. 1c), and eventually died between 14 and 36 weeks after birth (mean life span 18 weeks, Fig. 1i) with severe and sustained hyperglycaemia (>30 mmol l^−1^). Female βRapKO mice also became diabetic, but the phenotype developed more slowly and was less severe than in males (Supplementary Fig. 1b,c).

### Decreased beta cell mass in βRapKO mice

**Figure 2:**
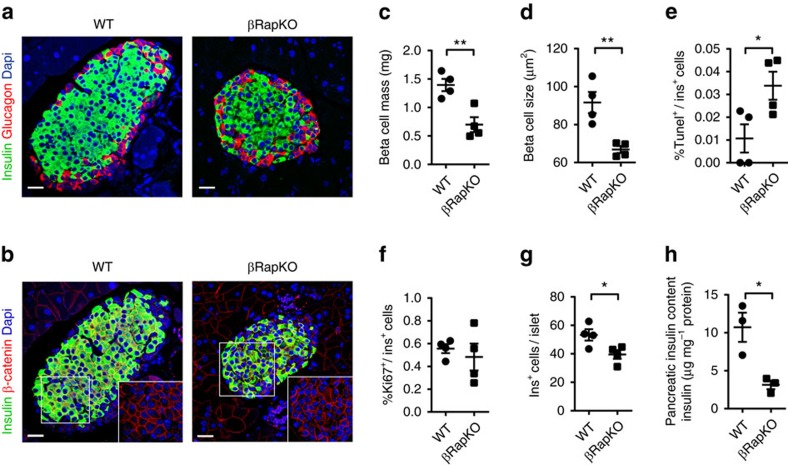
Lack of *Raptor* in beta cells leads to decreased beta cell mass. (**a**) Representative pancreatic sections immunostained with glucagon (red) and insulin (green) (*n*=4). Scale bars, 20 μm. (**b**) Representative pancreatic sections immunostained with β-catenin (red) and insulin (green) (*n*=4). Scale bars, 20 μm. (**c**,**d**) The beta cell mass (**c**) and the size (**d**) of individual beta cell in WT and βRapKO mice were determined (*n*=4). (**e**) The apoptosis of beta cell was detected by TUNEL assay and percentage of Tunel^+^insulin^+^ cells was calculated (*n*=4). (**f**) The proliferation of beta cell was determined by quantification the percentage of Ki67^+^insulin^+^ cells (*n*=4). (**g**) The number of insulin^+^ cells per islet was calculated (*n*=4). (**h**) Pancreatic insulin content normalized by protein concentration was shown (*n*=3). All the experiments were performed on 8-week-old WT and βRapKO mice. Results were presented as mean±s.e.m. of independent experiment indicated as above, *P<0.05, **P<0.01, unpaired Student's *t* test.

To understand if diabetes in βRapKO mice was caused by reduced beta cell mass, we examined islets morphology in 8-week-old WT and mutant mice. The 8-week-old βRapKO mice did not display disrupted islet structure, and their alpha cells were still residing at the periphery (Fig. 2a). Notably, the adjusted beta cell mass of βRapKO was 49.8% lower than WT (Fig. 2c). It is known that mTORC1 regulates beta cell growth^17^. To evaluate beta cell size, we used insulin staining to mark beta cells and β-catenin staining to highlight cell boundaries (Fig. 2b): a 27% reduction in beta cell size was observed in βRapKO mice (Fig. 2d). We detected a three-fold increase in the percentage of apoptotic Tunel^+^insulin^+^ cells (Fig. 2e), with comparable proportions of Ki67^+^insulin^+^ cells in mutant islets (Fig. 2f). These changes resulted in a significant decrease in the number of insulin^+^ cells per islet (Fig. 2g). Therefore, ablation of *Raptor* affected beta cell mass and number, possibly due to defects in beta cell growth and survival. Moreover, a striking reduction in pancreatic insulin content (Fig. 2h) in βRapKO mice further demonstrated that loss of functional beta cell mass was attributed to the poor glycemic control. We also checked the pancreata from 16-week-old βRapKO mice with prolonged severe hyperglycaemia (6-h fasted glucose, 23.8±1.4 mmol l^−1^). In this case, we found a change in alpha cell distribution, with some located in the centre of islets (Supplementary Fig. 2).

### Gene-expression profile of βRapKO islets

To understand the global molecular basis of the phenotype caused by the loss of *Raptor* in beta cells, we isolated pancreatic islets from 8-week-old βRapKO and WT mice. At this time point, βRapKO mice did not show weight loss and their overnight fasting glucose was below 8 mmol l^−1^. We compared gene-expression profile by microarray of islets between the two groups and detected significant differences in the expression levels of 1,150 genes (fold change>1.5), among which 786 were increased and 364 were decreased.

**Figure 3:**
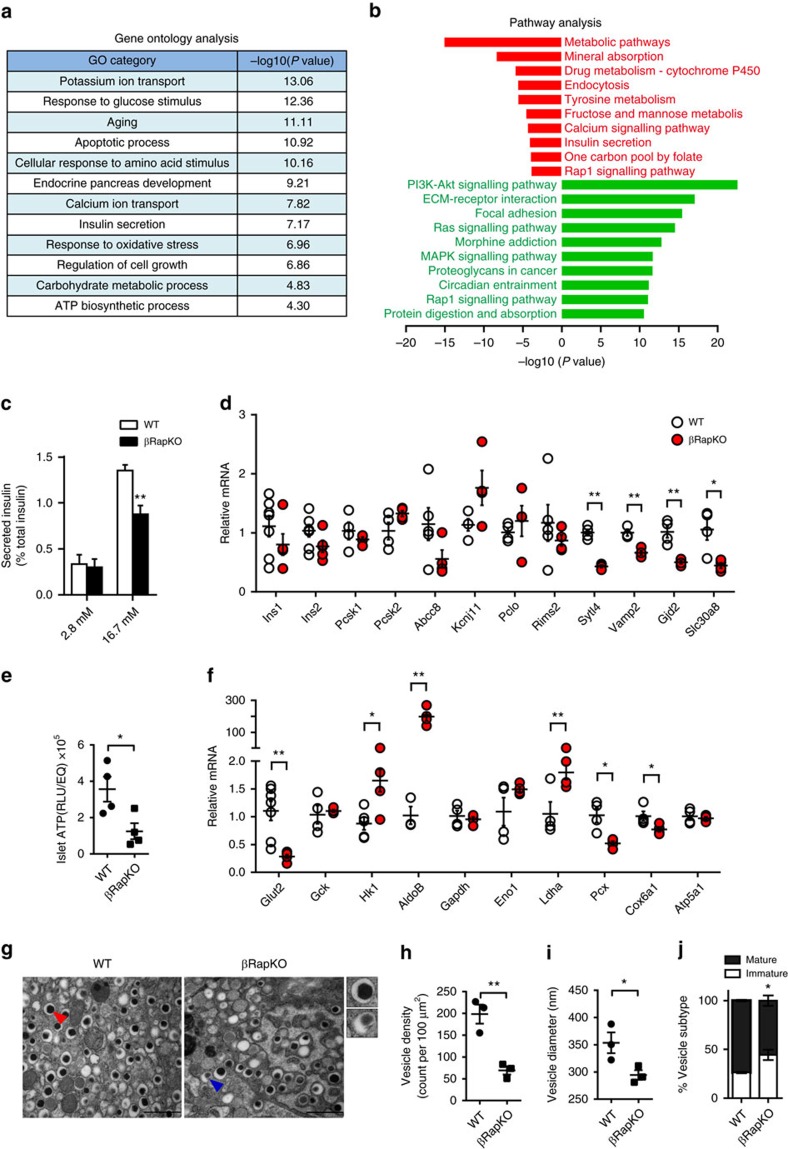
Loss of *Raptor* in beta cells impairs GSIS and ATP production. (**a**) GO analysis of differentially expressed genes as identified by microarray analysis of 8-week-old WT (*n*=5) and βRapKO (*n*=4) mice in associated with beta cell function. (**b**) Pathway analysis of top 10 increased (green) and decreased (red) pathways. (**c**) Isolated islets from 8-week-old WT and βRapKO mice were incubated at 2.8 and 16.7 mM glucose for 1-h. Secreted insulin was normalized to total insulin content in the islets (*n*=6). (**d**) RT-PCR confirmed genes involved in insulin biogenesis and secretion (*n*=3–5). (**e**) ATP content of islets from 8-week-old WT and βRapKO mice was determined (*n*=4). (**f**) RT-PCR confirmed genes involved in metabolism (*n*=3-5). (**g**) Transmission electron microscopy of pancreatic sections from 8-week-old WT and βRapKO mice (*n*=3). Red and blue arrowheads point to typical vesicles containing mature and immature insulin-dense core granules, respectively. Scale bars, 1 μm. (**h**–**j**) Analysis of ultrastructural beta cell (*n*=3), including quantification of (**h**) vesicle density, (**i**) vesicle diameter and (**j**) the percentage of mature (black bar) and immature vesicles (white bar). Results were presented as mean±s.e.m. of independent experiment indicated as above, *P<0.05, **P<0.01, unpaired Student's *t* test.

To define the cellular processes affected in βRapKO islets, we performed Gene Ontology (GO) analysis of differentially expressed genes. This analysis revealed alterations of several biological processes that are crucial to beta cell function, that is, insulin secretion, cell growth and apoptotic process, carbohydrate metabolic process, ATP biosynthetic process and ion transport following *Raptor* ablation (Fig. 3a). We further used the ingenuity pathway analysis to investigate alterations of known pathways and listed the top 10 increased and decreased pathways in βRapKO islets (Fig. 3b). Metabolic pathways, calcium signalling and insulin secretion were among the top decreased cellular biological processes, while PI3K-AKT, RAS and MAPK signalling related to canonical mitogenic and survival pathways were preferentially increased in mutant islets (Fig. 3b).

### Defects in GSIS and glucose metabolism in βRapKO islets

The observation that crucial pathways required for physiologic insulin secretion were preferentially decreased in βRapKO transcriptome led us to evaluate beta cell function *ex vivo*. In parallel to decreased pancreatic insulin content, a dramatic reduction in both insulin (58.9% of WT, Supplementary Fig. 3a) and protein content (71.8% of WT, Supplementary Fig. 3b) was observed in islets from 8-week-old βRapKO mice. By calculating insulin secretion as a percentage of total islet insulin content, we found that 8-week-old mutant islets secreted nearly 40% less of their total insulin than WT at 16.7  mmol l^−1^ glucose, whereas they had comparable basal insulin secretion at 2.8 mM glucose (Fig. 3c). Impaired GSIS was also observed when insulin secretion was corrected by total DNA content (Supplementary Fig. 3c). We then evaluated whether *Raptor* ablation also affects formation of insulin secretory vesicles. We analysed beta cell ultrastructure in 8-week-old βRapKO and WT using electron microscopy (Fig. 3g) and found a significant decline in the overall number (Fig. 3h) and mean size of secretory vesicles (Fig. 3i). Moreover, the proportion of immature vesicles was significantly increased in *Raptor*-deficient beta cells (Fig. 3j). We conclude that loss of *Raptor* results in a defect in protein/insulin production, GSIS response and secretory vesicle formation/maturation.

Interestingly, we did not observe differences in mRNA expression levels of *Ins1*, *Ins2* and proinsulin/insulin convertases (*Pcsk1*, *Pcsk2*) in 8-week-old βRapKO and WT islets (Fig. 3d). It is known that mTORC1 regulates translational initiation, which occurs primarily at the level of translation of pre-transcribed *Insulin* mRNA^31^. Indeed, deletion of *Raptor* induced dephohsphorylation of 4E-BP1 and S6 (Fig. 1b) and reduced the abundance of ribosomal proteins, that is, RPL7 and RPL26 (Supplementary Fig. 3e), indicating a diminished translational capacity. We then interrogated expression of genes implicated in insulin release, and found that most of them were not changed, including *Kcnj11*, *Abcc8*, *Pclo*, *Rims2* (Fig. 3d). In contrast, genes encoding proteins participating in granule trafficking and membrane exocytosis such as *Vamp2*, *Sytl4*, *Gjd2* were significantly decreased (Fig. 3d), as was the type 2 diabetes-associated gene *Slc30a8*, encoding zinc transporter-8 (*ZnT8*), which is important for insulin secretion^32^ (Fig. 3d).

In *Raptor*-deficient islets, metabolic pathway was the top decreased biological process identified by microarray analysis (Fig. 3b). To determine whether the failure to respond to glucose occurs at the level of glucose transport across the membrane, we examined *Glut2* levels in 8-week-old βRapKO mice and found a dramatic decrease in *Glut2* mRNA expression and protein levels, as determined by RT-PCR (Fig. 3f) and immunostaining (Supplementary Fig. 3f). Moreover, the key anaplerotic enzyme in mitochondria, pyruvate carboxylase (*Pcx*), as well as several other genes encoding subunits of electron transport chain complexes I–V including *Ndufs2*, *Ndufb8, Cox6a2*, *Cox6c*, *Cox8a*, *Cox8c*, *ATP5a1*, *ATP5j2* (Fig. 3f and Supplementary Table 1) were significantly decreased. Of interest, several glycolytic enzymes, including lactate dehydrogenase A (*Ldha*), aldolase B (*AldoB*) and hexokinase 1 (*Hk1*) that are minimally expressed in mature beta cells were significantly upregulated (Fig. 3f). We further confirmed that LDHA protein level was 4.25-fold increased by western blot (Supplementary Fig. 3g). These characteristics of enhanced glycolytic gene expression in βRapKO islets resemble those of poorly responsive neonatal islets^33^. Next, we measured intracellular ATP content and found that ATP level in 8-week-old mutant islets was decreased by 65% compared to WT (Fig. 3e). mTORC1 has been suggested to control mitochondrial oxidative function via its target, proliferator-activated receptor gamma coactivator 1 (PGC1α)^34^. Accordingly, we also detected a strong decrease in PGC1α protein abundance in the mutants (Supplementary Fig. 3e).

### Loss of beta cell functional maturity in βRapKO mice

**Figure 4:**
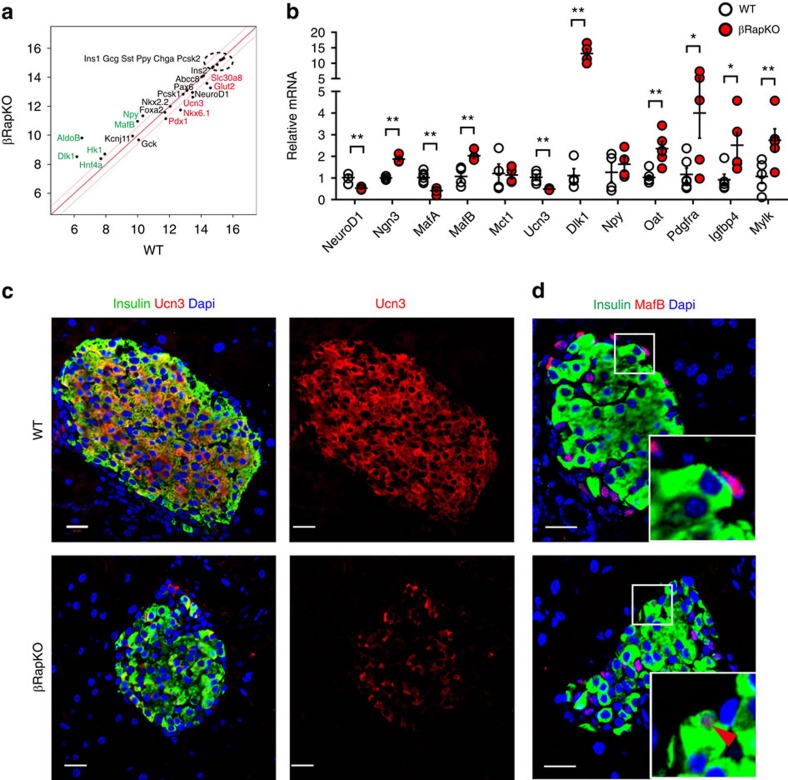
Depletion of *Raptor* in beta cells results in loss of functional maturity. (**a**) Scatter plots of expression levels of global genes critical for beta cell identity and maturity from microarrays of 8-week-old WT and βRapKO islets. Red lines mark a 1.5-fold difference in expression. Increased genes were marked in green, while decreased ones were in red: |FC|> 1.5, *P*<0.05. (**b**) RT-PCR analysis of transcription factors and genes involved in beta cell maturation (*n*=3–5). (**c**) Representative pancreatic sections immunostained with Ucn3 (red) and insulin (green) of 8-week-old mice (*n*=4). Scale bars, 20 μm. (**d**) Representative pancreatic sections immunostained with MafB (red) and insulin (green) of 8-week-old mice (*n*=4). Scale bars, 20 μm. Results were presented as mean±s.e.m. of independent experiment indicated as above, *P<0.05, **P<0.01, unpaired Student's *t* test.

Decreased expression of key metabolic genes *Glut2* and *Pcx*, derepression of glycolytic gene *Ldha*, *AldoB* and *Hk1*, as well as reduced ATP production and GSIS, are properties shared by 8-week-old βRapKO islets and fetal beta cells^35^,^36^,^37^,^38^. Importantly, *Raptor* ablation significantly reduced *Pdx1, Nkx6.1, NeuroD1* and *MafA* expression (Fig. 4a,b and Supplementary Fig. 3d), which are essential transcription factors for maintaining beta cell functional maturity^5^,^6^,^7^,^8^. Urocortin 3 (UCN3) is a peptide hormone expressed exclusively in mature beta cells and was recently identified as a molecular marker of functional beta cell maturation^14^. The level of *Ucn3* mRNA decreased >50% in islets from 8-week-old βRapKO mice (Fig. 4b). Accordingly, double immunofluorescence labelling demonstrated strong Ucn3 staining in nearly all insulin^+^ cells in 8-week-old WT, but remarkably decreased staining in mutant beta cells of age-matched animals (Fig. 4c). We found an increase in neonatal markers^36^ delta-like 1 homologue (*Dlk1*) and *MafB* expression in 8-week-old *Raptor*-deficient islets, with comparable transcriptional level of *Npy* (Fig. 4b). Moreover, we found that 3.8% of cells were insulin^+^MafB^+^ in 8-week-old βRapKO pancreas, but not in WT controls, as determined by morphometric analysis of immunostaining (Fig. 4d). Interestingly, expression of the endocrine progenitor marker *Ngn3* doubled in mutant islets. (Fig. 4b).

Ectopic expression of genes that are disallowed in beta cells is also crucial to maintaining the mature phenotype of beta cells^39^. Pullen *et al*.^40^ and Thorrez *et al*.^41^ have identified 39 and 33 disallowed genes, respectively, that are downregulated in islets relative to all other mouse tissues studied (red circles in Supplementary Fig. 5a). Remarkably, we found 31 out of 65 genes in 8-week-old βRapKO islets were significantly upregulated based on microarray data (blue circle in Supplementary Fig. 5a and listed in Supplementary Fig. 5b). Next, we confirmed the upregulation of platelet-derived growth factor receptor α (*Pdgfra*), insulin-like growth factor binding protein 4 (*Igfbp4*), ornithine aminotransferase (*Oat*) and myosin light chain kinase (*Mylk*) in 8-week-old *Raptor*-deficient islets by RT-PCR (Fig. 4b, Supplementary Fig 3d).

**Figure 5:**
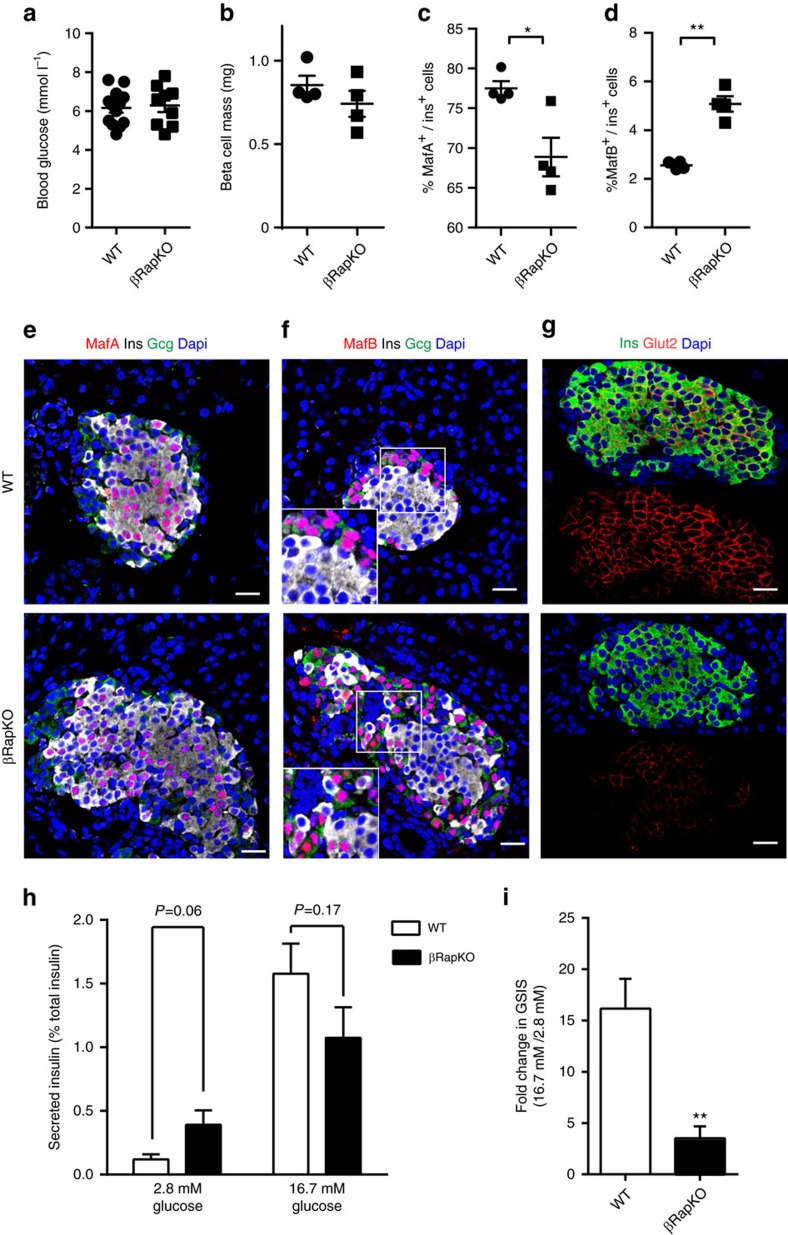
Functional immaturity is observed in beta cells from 2-week-old βRapKO mice. (**a**,**b**) Random blood glucose (**a**) and the beta cell mass (**b**) of 2-week-old WT and βRapKO mice (*n*=4). (**c**,**d**) Percentages of MafA^+^insulin^+^ (**c**) and MafB^+^insulin^+^ (**d**) cells were calculated (*n*=4). (**e**,**f**) Representative pancreatic sections from 2-week-old WT and βRapKO mice with triple staining: MafA (**e**) or MafB (**f**) (red), glucagon (green) and insulin (white) (*n*=4). Scale bars, 20 μm. (**g**) Representative pancreatic sections immunostained with Glut2 (red) and insulin (green) (*n*=4). Scale bars, 20 μm. Isolated islets from 2-week-old WT (*n*=5) and βRapKO (*n*=6) mice were incubated at 2.8 mM and 16.7 mM glucose for 1-h. (**h**) Secreted insulin was normalized to total insulin in the islets. (**i**) Stimulation index (fold change in GSIS) was calculated. Results were presented as mean±s.e.m. of independent experiment indicated as above, *P<0.05, **P<0.01, unpaired Student's *t* test.

To exclude the possibility that the observed immature phenotype is merely caused by hyperglycaemia, we performed experiments on 2-week-old euglycemic βRapKO mice (Fig. 5a) and WT with comparable beta cell mass (Fig. 5b). These young βRapKO mice exhibited similar beta cell proliferation rates and number of insulin^+^ cells per islet, but a significant decreased beta cell size, insulin content and increased beta cell apoptosis (Supplementary Fig. 4). We then performed immunostaining with antibodies against insulin, glucagon and MafA (or MafB) (Fig. 5e,f). Quantification revealed that the percentage of beta cells coexpressing MafA was decreased (Fig. 5c), while the proportion of insulin^+^MafB^+^ cells was significantly increased (Fig. 5d) in mutant islets. Moreover, a significantly lower level of GLUT2 expression was found in mutant beta cells, as determined by immunostaining (Fig. 5g). Importantly, isolated islets from 2-week-old mutant mice displayed an ‘immature' response to basal and stimulated glucose: they had elevated basal insulin secretion at 2.8 mM glucose, but reduced GSIS response under high glucose (16.7 mM) stimulation (Fig. 5h). Then we calculated their stimulation index (fold change in GSIS), and found that WT and mutant islets can be significantly distinguished (Fig. 5i). Thus, the changes in beta cells antedate the onset of diabetes and are likely a cause, rather than a result of it.

### Altered DNA methylation and disallowed genes in βRapKO islets

**Figure 6:**
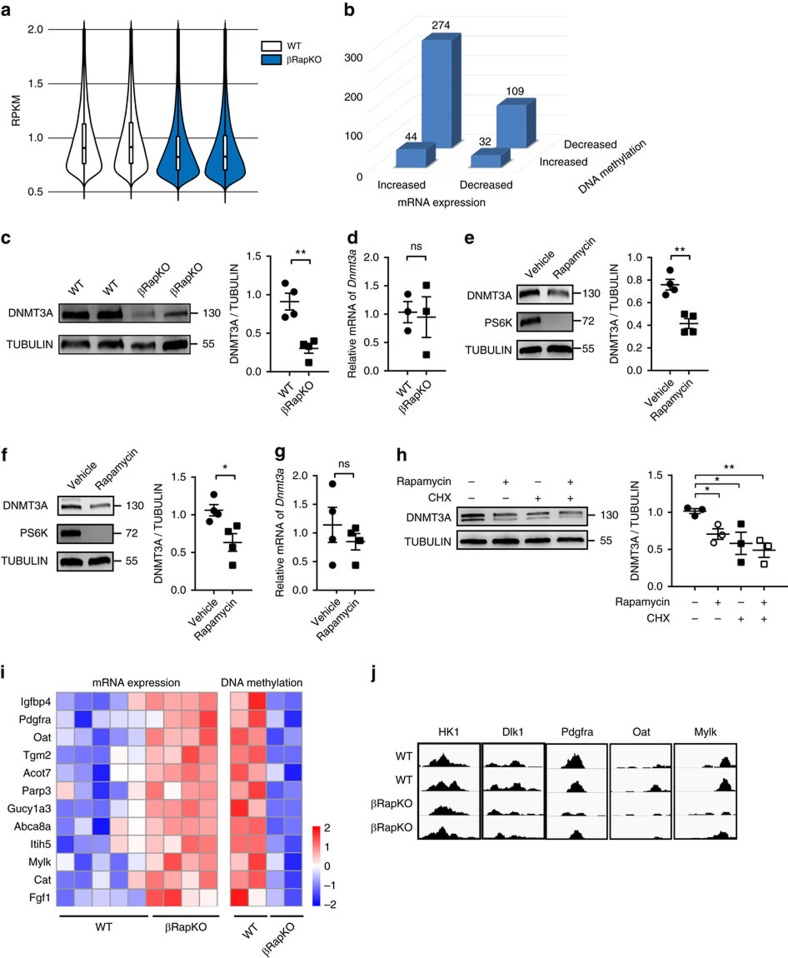
*Raptor* ablation in beta cells demethylates and derepresses disallowed genes. (**a**) The violin plots show methylation distribution based on genome-wide analysis of DNA extracted from 8-week-old WT and βRapKO islets. (**b**) The number of genes that exhibit both differential DNA methylation and gene expression in pancreatic islets from 8-week-old WT and βRapKO mice. (**c**) Western blot showed the expression level of DNMT3A in 8-week-old WT and βRapKO islets (*n*=4). (**d**) Relative mRNA expression level of *Dnmt3a* in 8-week-old WT and βRapKO islets (*n*=3). (**e**) Isolated islets were treated with 25 nM rapamycin for 48 h, and DNMT3A protein expression was assayed by immunoblot. (*n*=4). (**f**,**g**) INS-1 cells were treated with rapamycin for 48 h, (**f**) DNMT3A protein expression was assayed by immunoblot and (**g**) *Dnmt3a* mRNA levels were assayed by Q-PCR. (*n*=4). (**h**) INS-1 cells were treated in the presence or absence of rapamycin for 48 h, with or without CHX in the last 12 h incubation, and expression of DNMT3A was assayed by immunoblot (*n*=3). (**i**) Heat map of 12 disallowed genes obtained from microarrays and MeDIP-seq, with preferential change by 1.5-fold both at mRNA and DNA methylation levels. (**j**) Local methylation pattern of maturation related and disallowed genes. Results were presented as mean±s.e.m. of independent experiment indicated as above, *P<0.05, **P<0.01, unpaired Student's *t* test or ANOVA were used for multiple groups.

DNA methylation has been proposed to drive functional maturation of pancreatic beta cells^42^. To evaluate the possibility that altered methylation is involved in mTORC1-dependent maturation, DNA immunoprecipitation sequencing (MeDIP-seq) was carried out on islets isolated from 8-week-old βRapKO and WT mice. We showed distribution frequencies in violin plots, and we could clearly find that median, 25% quantile and 75% quantile of methylation values (RPKM) in βRapKO mice were all lower than WT islets (Fig. 6a). A total of 22,046 differential methylation regions (DMRs) were significantly changed (fold-change>1.5). Among them, 17,144 regions corresponding to 5,713 genes were hypo-methylated; while 4,902 regions corresponding to 1,783 genes were hyper-methylated. Decrease levels of DNA methylation have previously been associated with transcription upregulation^43^. We therefore sought to correlate changes in DNA methylation with changes in mRNA expression (fold-change>1.5) of the same genes and identified 1,224 DMRs related to 459 genes whose changes were consistent with changes in gene expression (Fig. 6b). A total of 306 genes had an inverse relation, with 90% exhibiting a decrease in DNA methylation and an increased mRNA expression (Fig. 6b), including many pancreatic beta cell disallowed genes (Fig. 6i).

It has been reported that loss of *Dnmt3a* in pancreatic beta cells induced *Ldha*, *Hk1*, *AldoB* demethylation and upregulation, thus prevents developmental metabolic reprogramming, resulting in loss of GSIS^42^. We found a comparable *Dnmt3a* mRNA expression, but a significant decrease in DNMT3A protein abundance in *Raptor*-deficient islets (Fig. 6c,d), as well as in intact islets and INS-1 cell *in vitro* treated with mTORC1 inhibitor rapamycin (Fig. 6e–g). As shown in Fig. 6h, 12 h treatment with protein translation inhibitor Cycloheximide (CHX) significantly reduced DNMT3A protein expression, to a similar extent as 48 h rapamycin did. Importantly, a further suppression of DNMT3A abundance did not occur when rapamycin treatment occurred in the presence of Cycloheximide (Fig. 6h). These results suggest that mTORC1 directly regulates DNMT3A levels via translational control.

Notably, our data showed that ∼40% of the upregulated disallowed genes in *Raptor*-deficient islets were found to be preferentially hypo-methylated (Fig. 6i). We confirmed that genes expressed in the neonatal period and beta cell disallowed genes (*Hk1*, *Dlk1*, *Pdgfra*, *Oat* and *Mylk*) were hypo-methylated (Fig. 6j) and transcriptionally activated (Fig. 4b) in *Raptor*-deficient islets. These results indicate that DNA methylation might participate in the control of repression of disallowed genes in βRapKO mice.

## Discussion

The surprising finding of our work is that ablation of *Raptor* in adult beta cells resulted in a loss of functional maturation. Although it is well known that functional maturation is important for proper beta cell function, pathways that regulate this process are virtually unknown. By demonstrating a lag in functional maturation in *Raptor*-deficient beta cells, our data not only begin to provide insight into this complex process, but also raise the possibility of engaging the TOR pathway to induce beta cell maturation *in vivo*. Moreover, lack of functional maturation has consistently plagued efforts to develop glucose-responsive beta cell from ES or iPS cells. Our data provide a new mechanism that can be leveraged to induce maturation *in vitro*.

mTORC1 is known to be essential for beta cell function and mass^44^; however, which aspects of the maintenance of functional beta cell mass require mTORC1 remains poorly understood. In the present study, we generated βRapKO mice in which mTORC1 signalling is inactivated in beta cells at the onset of insulin gene expression. These mice displayed defective insulin secretion in response to glucose and overt diabetes starting at the age of 4 weeks. Morphometry showed that the decreased GSIS capacity could be partly attributed to a significant reduction in beta cell mass, accompanied by diminished beta cell size and number. Another remarkable finding of the present study is that pancreas and islet from βRapKO mice contained less than half the normal amount of insulin. The total protein concentration in islets was also reduced by 30%. Impaired efficacy of protein synthesis is also found in *Raptor*-deficient skeletal muscle, resulting in muscle dystrophy^28^. It is known that mTORC1 regulates protein synthesis through translational mechanisms^31^. Indeed, the reduced insulin production of *Raptor*-deficient islets was mainly due to post-transcriptional regulation, as evidenced by limited translational initiation and ribosomal biogenesis. Importantly, as early as 14 days after birth, mutant mice showed defects in insulin production, beta cell growth and survival before the onset of diabetes, indicating that mTORC1 directly regulates these functional characteristics, eventually leading to a loss of functional beta cell mass.

Our RNA profiling analysis identified signature abnormalities of insulin secretion and metabolic pathways in βRapKO islets. Islets from βRapKO mice secreted similar amount of insulin as WT at 2.8 mM glucose, but their GSIS was significantly lower. In mature beta cells, glucose is transported via GLUT2 and generates ATP in mitochondria, thus causing closure of ATP-sensitive K^+^ channels, ultimately leading to Ca^2+^ influx and insulin exocytosis^45^. We showed that the reduction of GSIS capacity in *Raptor*-ablated islets was not only caused by beta cell loss, but might also due to loss of GLUT2 expression, reduced expression of key cataplerotic enzyme *Pcx* and several components of OxPhos, impaired cellular ATP production, and defects in dense-core granule formation and maturity. Interestingly, mutant islets exhibited a metabolic profile resembling that of neonatal islets, in which glycolysis predominates and results in impaired GSIS^46^. Likewise, we found an induction of a number of glycolytic genes, including *AldoB*, *Hk1* and *Ldha*. The ectopic expression of *Hk1* and *Ldha* in beta cells has been reported to impair glucose metabolism and GSIS^47^,^48^. In addition, aberrant expression of *Hk1* in human beta cells has been associated with a form of congenital hyperinsulinemia^49^.

Evidence for a role of Raptor in functional maturation is manifold. First, in *Raptor*-deficient islets we found a significant decrease in *NeuroD1* and *MafA*, loss of which has been reported to reverse beta cell maturation^7^,^8^. Second, Urocortin3 (Ucn3), a molecular marker for functional beta cell maturation^14^, was significantly decreased at mRNA levels in *Raptor*-deficient islets. Immunofluorescence staining further confirmed that Ucn3 was highly expressed in all adult beta cells in WT, whereas the signal intensity of this protein dramatically declined in insulin^+^ cells from βRapKO mice. Third, transcripts encoding *Ngn3* and *Dlk1*, which are abundantly expressed in fetal and neonatal beta cells^36^,^50^,^51^ were strongly induced in adult βRapKO beta cells. Fourth, in 8-week-old mutant mice we observed that up to ∼4% of insulin^+^ cells maintained MafB expression, a feature of neonatal beta cells^50^,^52^. During embryonic stem cell differentiation, the transition from MafB to MafA is critical for beta cell functional maturation^52^. Notably, the decrease of insulin^+^MafA^+^ cells and the increase of insulin^+^MafB^+^ cells preceded the onset of diabetes, consistent with a causative role in hyperglycaemia, rather than a consequence thereof. Likewise, Glut2 was dramatically lowered in beta cells from euglycemic mutant animals. Moreover, P14 *Raptor*-deficient islets had a lower threshold for GSIS, secreting insulin in response to a lower glucose concentration than their age-matched WT islets, which is another feature of functional immaturity^14^. These progenitor-like beta cells showed a three-fold increase in apoptotic rate, implying that they underwent programmed cell death before achieving full maturation.

Equally important for normal beta cell function is the selective repression of a number of disallowed genes compared to other tissues^40^,^41^. The repression of these genes is critical in the context of maintaining mature beta cell phenotype, and expression levels of a number of these genes increase in animal models of type 2 diabetes^53^,^54^,^55^. Strikingly, our RNA profiling data showed that nearly 50% of the disallowed genes identified by Pullen *et al*.^40^ and Thorrez *et al*.^41^ were strongly induced in *Raptor*-deficient beta cells. Recent evidence suggests that DNA methylation directs beta cell functional maturation via modulating *Hk1* and *Ldha* expression^42^. In βRapKO islets, we detected a dramatic decrease in DNMT3A protein abundance, with comparable *Dnmt3a* mRNA expression. Rapamycin treatment also reduced DNMT3A protein abundance in isolated islet and INS-1 cells, which is primarily due to translational modulation. MeDIP-seq analysis revealed that *Raptor*-ablated islets had a significant decline in the average methylation level aggregated over all covered CpG sites. A decrease level of DNA methylation has previously been associated with transcriptional activation^43^. Approximately 40% of disallowed genes that were preferentially upregulated in βRapKO islets displayed hypo-methylation. Several of these demethylated genes, such as *Oat*^56^, acyl-CoA thioesterase 7(*Acot7*)^57^ and *Mylk*^58^ have the potential to interfere with glucose metabolism and insulin secretion. Demethylation and upregulation of *Pdgfra*, *Hk1* and *Dlk1* in βRapKO islets are associated with the process of beta cell maturation. Recent work showed that the progressive downregulation of *Pdgfra* during islet maturation is responsible for the age-dependent decrease in the proliferation capacity of islet cells^59^, whereas upregulation of *Pdgfra* was observed in islets in human type 2 diabetes and in a rat model of obesity-induced diabetes^54^,^55^. Moreover, *Hk1* is selectively excluded from beta cells and liver^40^ and its repression allows beta cell to sense glucose concentrations in the physiological range (>4 mM). The transient overexpression of *Hk1* in Min6 cells^60^ and primary beta cells^61^ yields an immature-like beta cell phenotype with enhanced glucose threshold for insulin secretion, increased basal glycolytic flux, elevated basal insulin release, and poor responsiveness to elevated glucose^14^. In the present study, we did not detect significant differences in the methylation levels of *Ldha* and *AldoB* in βRapKO islets, indicating that other regulatory mechanisms, such as microRNAs and histone modifications^62^,^63^,^64^, may be responsible for their induction. Currently, the function and the regulation of most of the disallowed genes are not fully understood. It is also not clear how much these deeply repressed genes contribute to the acquisition of properties required for beta cell functional maturation. Targeted *in vivo* rescue studies are needed to understand the extent and specific aspects of DNA methylation regulation that contribute to *Raptor* ablation-induced immature phenotype in the future.

**Figure 7:**
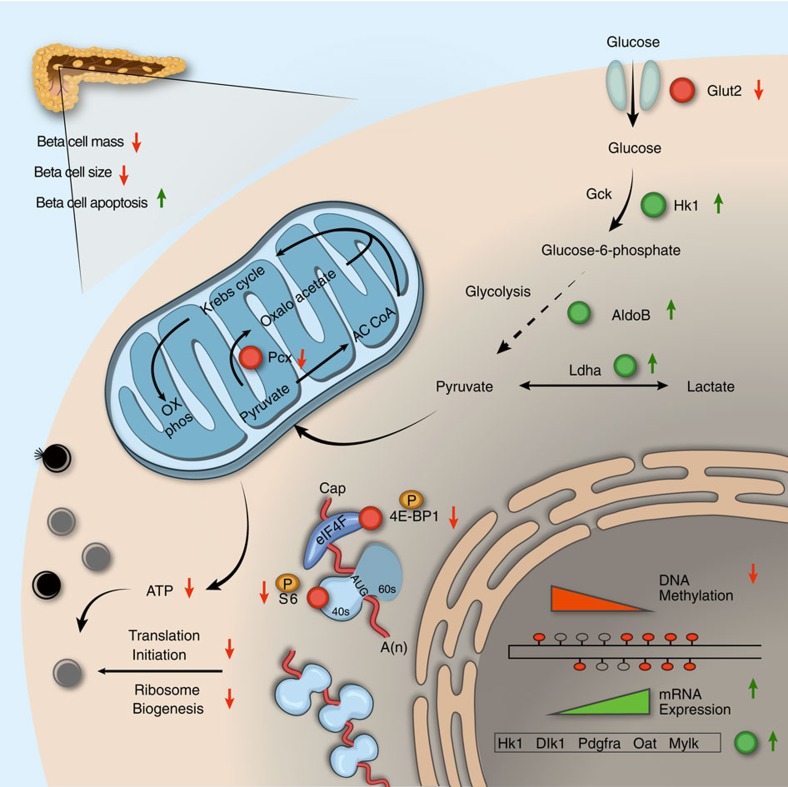
Raptor/mTORC1 function in adult beta cells. Raptor/mTORC1 directly regulates beta cell size and survival, and thus controls beta cell mass. Reduced expression of *Raptor* affects beta cell functional maturation in three aspects: First, reduced glucose uptake and metabolism (*Glut2* and *Pcx*) diminishes ATP production, leading to impaired insulin secretion via the stimulus-secretion coupling pathway. Second, inhibition of translation initiation and ribosomal biogenesis (possibly via dephosphorylation of 4E-BP1 and S6) severely impairs production of mature insulin and increases an overabundance of immature secretary vesicles. Third, decreased DNA methylation and increased expression of beta cell specific disallowed genes (that is, *Hk1*, *Dlk1*, *Pdgfra*, *Oat*, *Mylk*), might participate in the control of beta cell functional maturation.

This work provides support for the evolving concept that mTORC1 signalling not only controls beta cell mass, but also is critical for achieving beta cell functional maturation (Fig. 7). Our findings show that *Raptor* coordinates increased oxidative metabolism and translational capacity to ensure that beta cells acquire the mature GSIS phenotype. Epigenetic regulation, including DNA methylation, might participate in derepression of disallowed gene, which play a role in mTORC1-induced immaturity. Modulation of mTORC1 activity, or its regulatory pathways in beta cells might serve as therapeutic targets to protect beta cell function and create functional mature beta cells suitable for transplantation.

## Methods

### Animal

We used Cre/loxP system to conditionally delete *Raptor* in pancreatic beta cells. Beta cell specific *Raptor* knockout mice (βRapKO) were generated by crossing *Raptor*^*flox/flox*^ mice (purchased from the Jackson Laboratory, C57BL/6J) with mice expressing Cre recombinase driven by the rat insulin promoter (RIP-Cre, mixed C57BL/6J:129/SvJ), as described before^29^,^65^. βRapKO (*Raptor*^*flox/flox*^ RIP-Cre), βRapHET *(Raptor*^*flox/w*^ RIP-Cre) mice were used for experiments and their littermates (*Raptor*^*flox/flox*^) were used as WT control. Mice were fed *ad libitum* and housed in facility on a 12-h light-dark cycle. Genotypes of all mice were determined by PCR analysis of genomic DNA extracted from tail. In the current study, male mice were used in all the experiments, unless otherwise stated in the text. All animal experiments were performed in accordance with the principles of Guide for the Care and Use of Experimental Animals of Shanghai Jiao Tong University School of Medicine and approved by the Animal Care Committee of Shanghai Jiao Tong University.

### Metabolic studies

Weight and blood glucose of βRapKO, βRapHET and WT were monitored biweekly from 2 to 18 weeks. Fasting plasma insulin was measured at the age of 4, 8, 12 and 16 weeks. Intraperitoneal glucose tolerance test (IPGTT) and GSIS *in vivo* were performed on 8-week-old mice. Mice were injected intraperitoneally with 2 g dextrose/kg body weight after an overnight fasting. Blood glucose concentrations were measured at various time points (0, 15, 30, 60, 120 min) by glucometers (Accu-Chek, Roche, Mannheim, Germany). Blood samples were taken at 0, 15, 30 min after dextrose injection, and serum insulin concentrations were determined by using ELISA assay (Mouse Ultrasensitive Insulin ELISA kit, Alpco)^21^.

### Islet isolation and *ex vivo* experiments

Pancreatic islets were isolated, as described before^66^. Briefly, mice were injected with Collagenase P (Roche) solution into the common hepatic bile duct to perfuse the pancreas. Pancreas were then removed and digested at 37 °C. Islets were handpicked after several purification steps under a microscope. The ATP content of isolated islets was measured using an assay kit (FLASC; Sigma) and expressed as relative light units per islet equivalent using a luminometer (Promega, Madison, WI).

For *ex vivo* GSIS, isolated islets were recovered in 1640 RPMI supplemented with 10% serum, 11 mM glucose (islets from 8-week-old mice), or 2.8 mM glucose (islets from 2-week-old mice) for 4-h, followed by incubation with Krebs Ringer Bicarbonate HEPES solution (KRB) containing 2.8 mM glucose for 1-h. A total of 30 islets of similar size were randomly chosen and incubated at 2.8 or 16.7 mM glucose for 1-h. At the end of incubation, the insulin concentrations of the supernatant and insulin content were calculated. Islet insulin content was measured by insulin ELISA assay (Mouse Insulin ELISA kit, Alpco) and normalized by total DNA extracted using DNeasy Micro kit (Qiagen, GmBH, Germany).

For *in vitro* experiments, INS-1 cells (passage 23–35) were purchased from the CAMS Cell Culture Center (Beijing, China), which have been authenticated and tested for mycoplasma contamination. INS-1 cells were grown in RPMI 1640 medium containing 11.1 mM glucose and supplemented with 10 mM HEPES, 10% FBS, 1 mM L-glutamax, 1 mM sodium pyruvate, 50 μM 2-β-mercaptoethanol, 100 IU ml^−1^ penicillin and 100 μg ml^−1^ streptomycin at 37 °C in a humidified 5% CO_2_ atmosphere. The cells were sub-cultured, when they reached 80% confluence. After passaging, cells were synchronized in a medium containing 11.1 mmol l^−1^ glucose with or without 25 nM rapamycin (Cell Signaling Technology, Boston, MA, USA) for 48 h, 5 μM CHX (Sigma-Aldrich, St. Louis, MO, USA) was added in the last 12 h incubation. At the end of culture, cells were collected for protein or RNA extraction.

### Immunohistochemistry and Morphometric analysis

Pancreata were dissected, fixed and processed, as described before^21^. For immunofluorescence, sections of pancreata were stained with guinea pig anti-insulin (1:800, DAKO), rabbit anti-β-catenin (1:400, Cell signaling), mouse anti-glucagon (1:1,000, Abcam), rabbit anti-Ucn3 (1:500, Phoenix Pharmaceuticals), rabbit anti-MafB (1:200, Bethyl), rabbit anti-Glut2 (1:1,1000, Millipore), rabbit anti-MafA (1:200, Bethyl), rabbit anti-pS6 (Ser240/244) (1:200, Cell signaling). Detection was performed using Alexa Fluor 488, 594 and 647 (Jackson ImmunoResearch or Life Technologies). Images were captured by using an Olympus Microscope or Zeiss LSC 710 confocal microscope. Beta cell size was determined by measuring the internal area of insulin^+^ cells, as defined by β-catenin labelling using ImageJ software (National Institutes of Health, Bethesda, MD, USA), at least 500 beta cells per animal were measured. The beta cell proliferation was calculated using the percentage of Ki67^+^insulin^+^ cells, at least 2,500 beta cells per animal were counted. For beta cell apoptosis, Tunel^+^insulin^+^ cells were calculated and at least 4,000 beta cells per animal were counted. To obtain the number of beta cell per islet, ∼50 islets were imaged per animal.

To measure beta cell mass, pancreas was removed from mice, weighed, and then embedded and sectioned continuously. At least 10 evenly 200 μm apart sections throughout the entire pancreas were picked to perform immunostaining of insulin, followed by peroxidase conjugated secondary antibody, visualized using a DAB Peroxidase Substrate Kit (Fuzhou Maixin Biotech, China), and counterstained with eosin^66^. Digital images of whole pancreas were captured by a Nikon MZ 100 microscope. Total pancreatic and insulin positive areas of each section were measured using Meta-Morph version 6.1 (Molecular Devices). Beta cell mass was obtained by multiplying the ratio of total insulin positive area to total pancreatic area with the pancreas weight.

### RT-PCR

Total islet RNA was extracted using RNeasy Plus Micro kit (Qiagen, GmBH, Germany) according to the manufacturer's protocol and used for reverse transcription with Superscript III (Life technologies, Carlsbad, CA, USA) with random hexamer primers. Quantitative PCR amplification and detection were performed with SYBR Premix Ex Taq Mixes (TaKaRa, Kyoto, Japan) on a LightCycler 480 instrument (Roche Applied Science, Mannheim, Germany). The gene expression levels were normalized to individual β-actin. Primer sequences can be obtained in Supplementary Table 2.

### Microarray analysis

Total RNA was extracted from freshly isolated islets of 8-week-old βRapKO and WT mice using RNeasy Micro kit (Qiagen, GmBH, Germany) and sample qualities were assessed using an Agilent Bioanalyzer 2100 (Agilent technologies, Santa Clara, CA, USA), taking a minimal cut-off RIN >=7. We included at least 4–5 independent biological replicates, each composed of RNA isolated from islets of 5 animals with the same genotype. 2 μg of total RNA samples of each group were then used to generate biotinylated cRNA targets for the Affymetrix Gene Chip Mouse Genome 430.2.0 Array. Data were analysed using Genespring software (Agilent).

### MeDIP sequencing

Genomic DNA from freshly isolated islets was extracted using DNeasy Blood Tissue Kit (Qiagen, GmBH, Germany). MeDIP was performed using a MagMedIP kit (Diagenode, Denville, NJ, USA), as previously described^67^. Libraries were prepared following the manufacture's instruction and were sequenced by Illumina HiSeq 2500 with 50 cycles. Reads were aligned to reference mouse genome (mm10) by Bowtie2 v2.2.5 after QC and further pre-procession. Peaks were called by MACS v0.14.2, and DMRs (Differential methylation region) were analysed by MEDIPS v1.12.0R Bioconductor package. DMRs were calculated using a window size of 500 bp, extend by 100 bp, increasing in 500 bp increments, a minimum number of 10 reads in a window was required prior to inclusion in the DMRs. Additionally, DMRs were filtered out if it has no overlap with peaks called by MACS.

### Western blot

The freshly isolated islets were lysed, quantified, blotted and developed as described before^21^. Primary antibodies are listed as following: rabbit anti-RAPTOR (1:1,000, Cell Signaling), rabbit anti-PS6 (Ser240/244) (1: 1,000, Cell Signaling), rabbit anti-4E-BP1 (1: 1,000, Cell Signaling), rabbit anti-PS6K (Thr389) (1:1,000, Cell Signaling), mouse anti-DNMT3A (1: 2,000, Novus Biologicals), rabbit anti-RPL7 (1: 1,000, Bethyl), rabbit anti-RPL26 (1: 1,000, Bethyl) and rabbit anti-LDHA (1: 1,000 Abcam), rabbit anti-PGC1a (1: 1,000 Abcam), mouse anti-TUBULIN (1: 20,000, Sigma-Aldrich). TUBULIN was used as an internal control to normalized band intensity.

### Electron microscopy

Samples were fixed, dehydrated, stained and embedded as previously described^68^. The density and subtype of beta cell vesical were determined by counting images captured at × 7,400 magnification^68^. The vesical size was measured by analysing images captured at × 16,500 magnification^68^. Each analysis was based on at least eight images per mice.

### Statistics

No randomization was used in this study, and the investigators were not blinded to group allocation during experiments or outcome assessments. No statistical method was used to predetermine sample size. Sample sizes were estimated from previous experience and common knowledge of animal studies. All data are presented as mean±s.e.m. Statistical analysis was performed with unpaired Student's *t* test with a two-tailed distribution for two groups or ANOVA for multiple groups. *P*<0.05 was considered as statistically significant.

### Data availability

Gene expression and MeDIP-seq data that support the findings of this study have been deposited in NCBI GEO (Gene Expression Omnibus) under accession code GSE84404 and GSE95775. All data supporting the findings of this study are available from the corresponding author on reasonable request.

## Additional information

**How to cite this article:** Ni, Q. *et al*. Raptor regulates functional maturation of murine beta cells. *Nat. Commun.* **8,** 15755 doi: 10.1038/ncomms15755 (2017).

**Publisher's note:** Springer Nature remains neutral with regard to jurisdictional claims in published maps and institutional affiliations.

## Supplementary Material

Supplementary Information Supplementary figures and supplementary tables.
