# Supplementary material for: Raptor regulates functional maturation of murine beta cells
Source: Nat Commun. 2017 Jun 9;8:15755. doi: 10.1038/ncomms15755 (PMC5472774; doi:10.1038/ncomms15755)
Supplement: Supplementary Information — Supplementary figures and supplementary tables. [file ncomms15755-s1.pdf]

Supplementary information

Supplementary Figure 1.

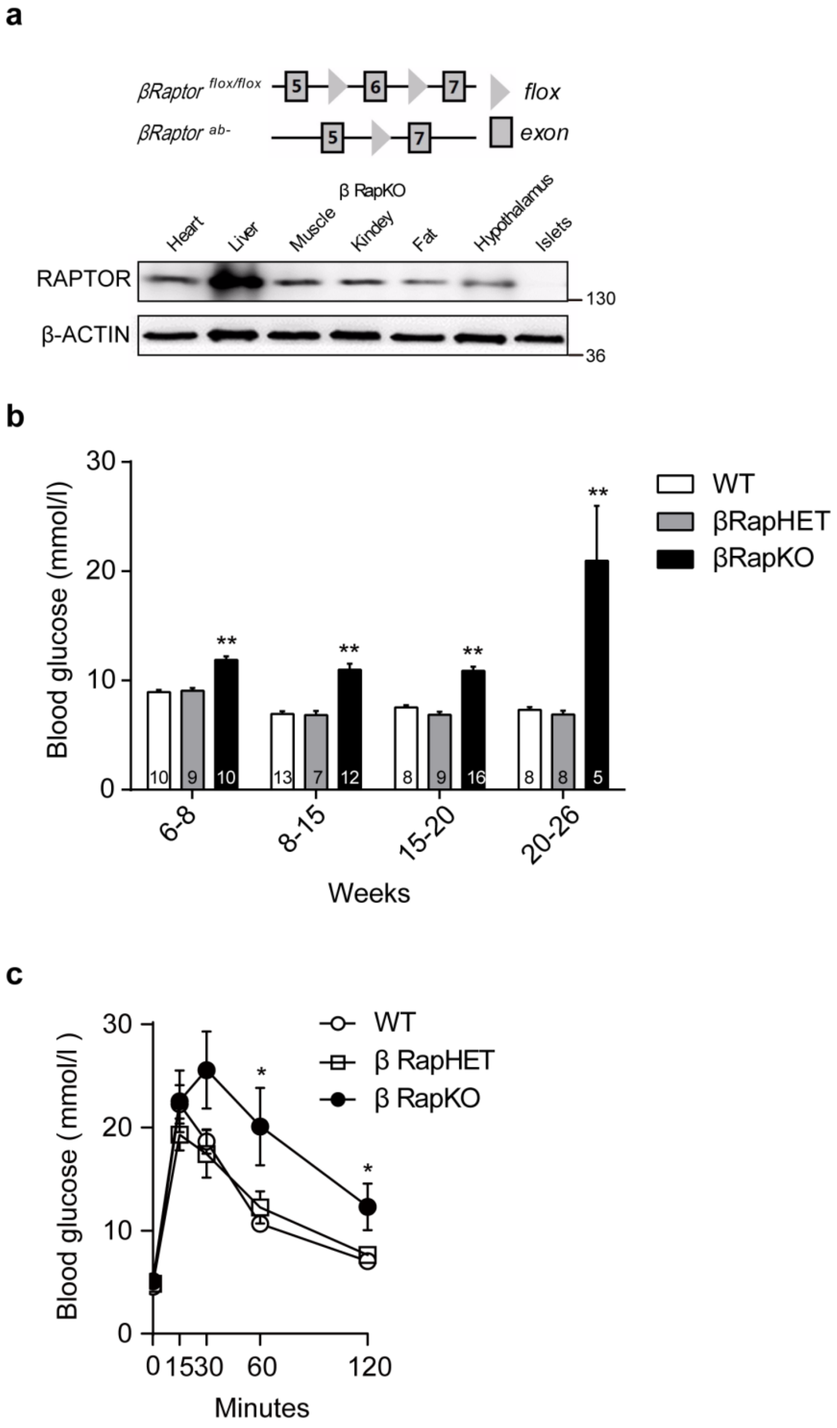

Supplementary Figure 1. Generation and validation of βRapKO mice.
(**a**) Western blot showed a restricted and significant reduction of RAPTOR in islets of βRapKO mice (n=3). (**b**) Random blood glucose of WT, βRapHET and βRapKO female mice at indicated time (n values in each bar). (**c**) Intraperitoneal glucose tolerance test was performed on 8-week-old female WT (n=4), βRapHET (n=5) and βRapKO mice (n=3). Results were presented as mean ± s.e.m. of independent experiment indicated as above, $*P < 0.05$; $**P < 0.01$, unpaired Student’s *t* test for two groups or ANOVA for multiple groups.

Supplementary Figure 2.

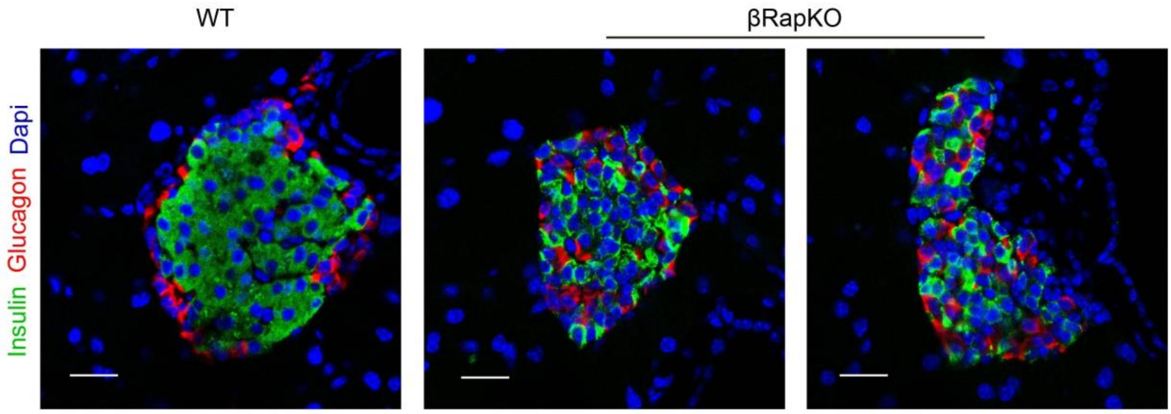

Supplementary Figure 2. Islet morphology of 16-week-old βRapKO mice.

Immunofluorescence staining for glucagon (red) and insulin (green) in 16-week-old WT and βRapKO pancreata (n=3). Scale bars, 20μm.

Supplementary Figure 3.

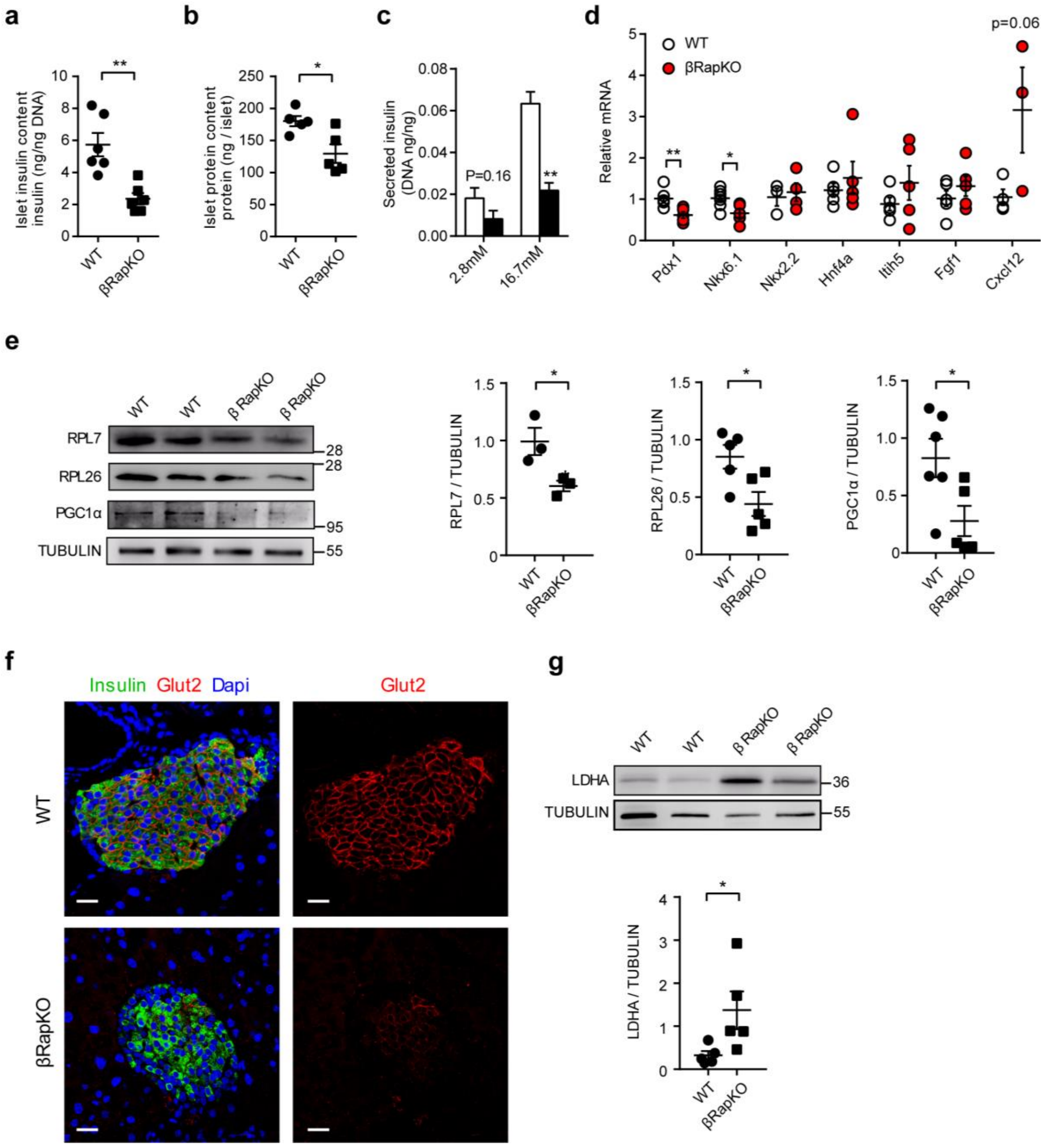

Supplementary Figure 3. βRapKO mice showed decreased ribosome biogenesis and functional maturity.

(**a**) Insulin content normalized to DNA content from lysates of islets isolated from 8-week-old WT and βRapKO mice (n=6). (**b**) Protein content was measured from lysates of islets isolated from 8-week-old WT and βRapKO mice (n=5). (**c**) Isolated islets from 8-week-old WT and βRapKO mice were incubated at 2.8mM and 16.7mM glucose for 1-h. Secreted insulin was normalized to DNA extracted from islets (n=6). (**d**) Genes confirmed by RT-PCR in microarray (n=3-5). (**e**) Western blot showed the expression level of ribosomal protein L7 (RPL7), ribosomal protein L26 (RPL26) and PGC1α in 8-week-old WT and βRapKO islets. Band intensities of RPL7, RPL26 and PGC1α normalized for the corresponding TUBULIN intensity were calculated (n=3-5). (**f**) Immunofluorescence staining for Glut2 (red) and insulin (green) in 8-week-old WT and βRapKO pancreata (n=4). Scale bars, 20μm. (**g**) Western blot showed the expression level of LDHA in 8-week-old WT and βRapKO islets. Band intensities of LDHA normalized for the corresponding TUBULIN intensity were calculated (n=4). Results were presented as mean ± s.e.m. of independent experiment indicated as above, **P* < 0.05; ***P* < 0.01, unpaired Student's *t* test.

Supplementary Figure 4.

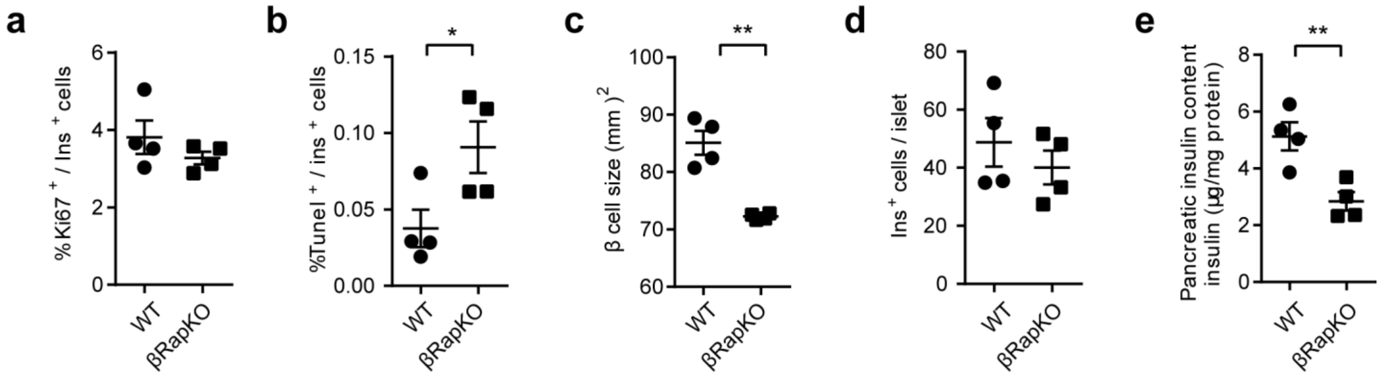

Supplementary Figure 4. Islet morphology analysis of 2-week-old βRapKO mice.

(**a**) The proliferation of beta cell was determined by quantification the percentage of Ki67^{+}insulin^{+} cells (n=4). (**b**) The apoptosis of beta cell was detected by TUNEL assay and percentage of Tunel^{+}insulin+ cells was calculated (n=4). (**c**) The cell size of individual beta cell in WT and βRapKO mice was determined (n=4). (**d**) The number of insulin^{+} cells per islet was calculated (n=4). (**e**) Pancreatic insulin content normalized by protein concentration was shown (n=4). Results were presented as mean ± s.e.m. of independent experiment indicated as above, **P* < 0.05, ***P* < 0.01, unpaired Student's *t* test.

Supplementary Figure 5.

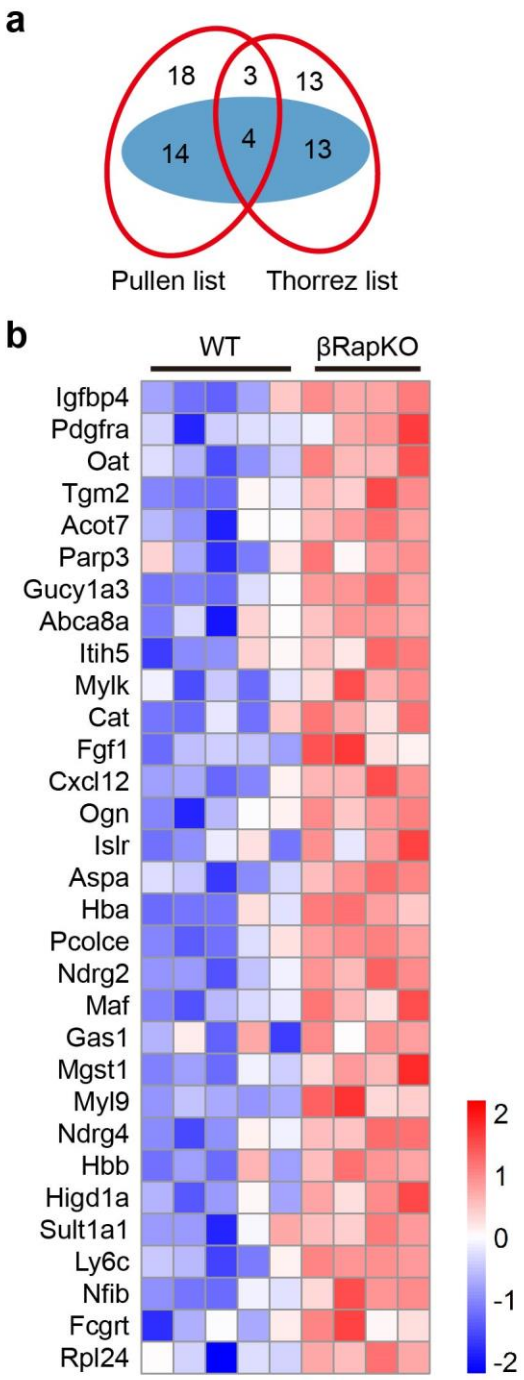

Supplementary Figure 5. Relatively expression of disallowed genes in βRapKO mice.
(**a**) Venn diagram showing the overlap between βRapKO upregulated genes (blue panel, 31 genes) and disallowed genes identified in the Thorrez et al and Pullen et al studies. (**b**) Heat map of 31 disallowed genes showing mRNA expression obtained from microarrays.

Supplementary Figure 6.

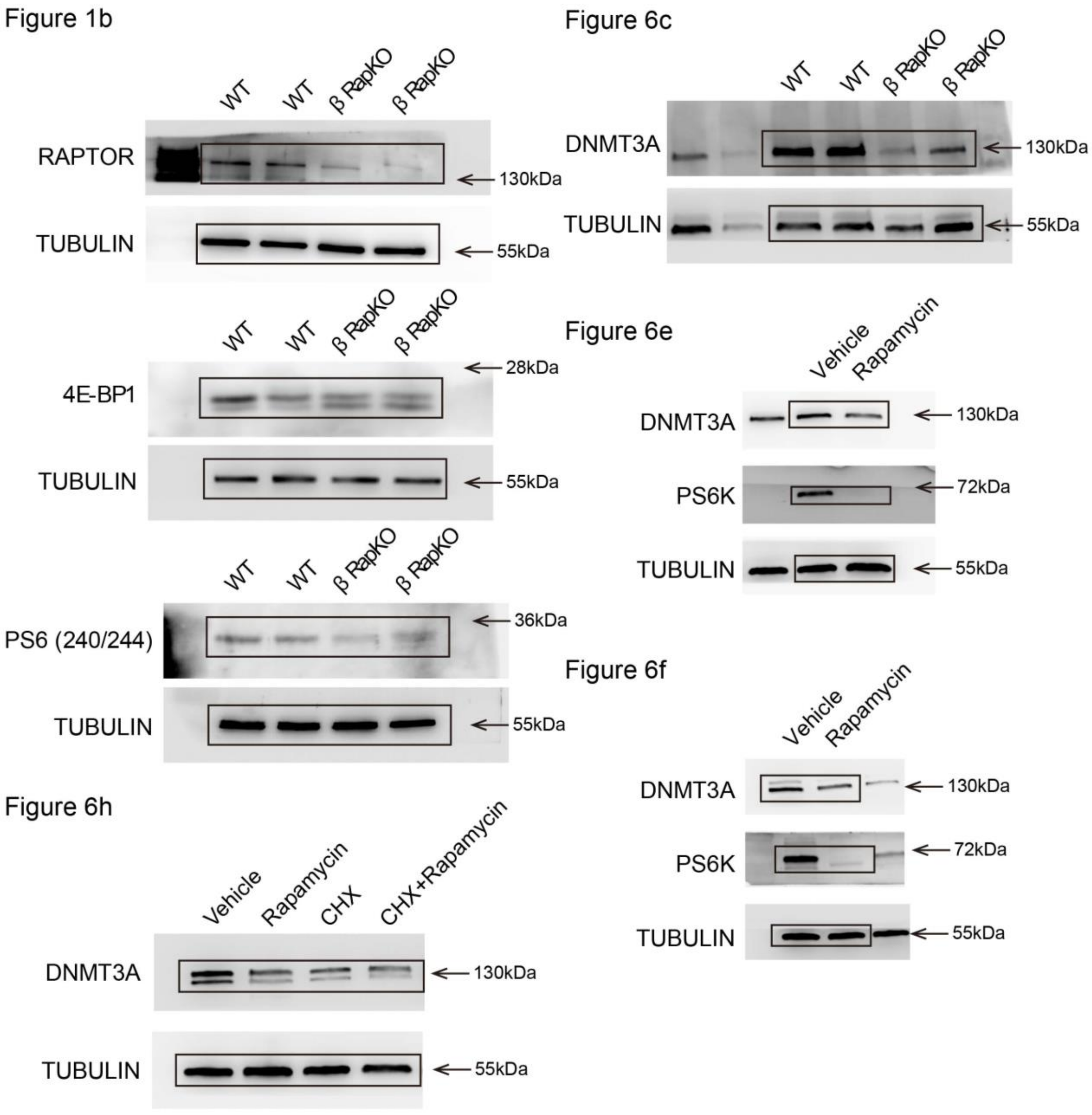

Supplementary Figure 6. Full-length western blots for Fig. 1b, Fig. 6c, e, f and h.

Supplementary Figure 7.

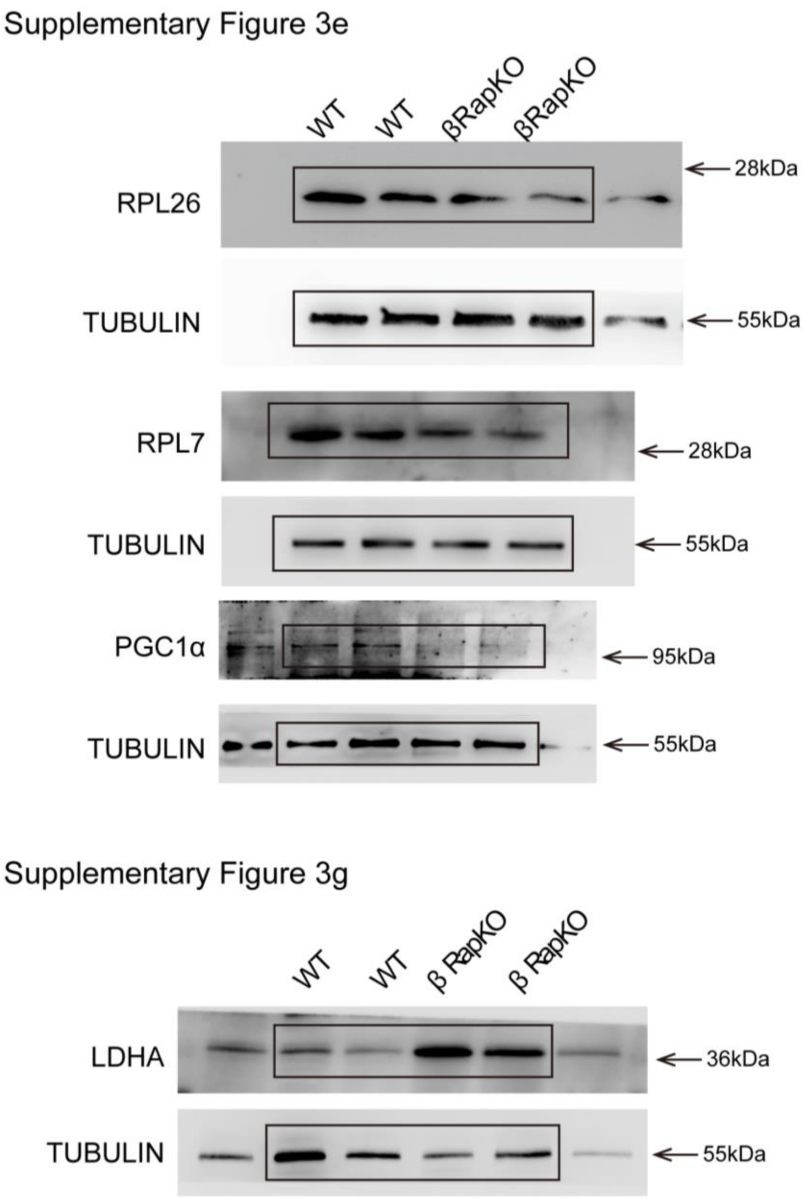

Supplementary Figure 7. Full-length western blots for Supplementary Figure 3e and Supplementary Figure 3g.

Supplementary Table 1.

List of metabolic genes expressed in 8-week-old βRapKO islets.

Probe_ID	Gene symbol	Fold change	*p* values
**Glycolysis**			
1449067_at	Slc2a2 (GLUT2)	0.40	0.00
1415802_at	Slc16a1(MCT1)	1.39	0.09
1437974_a_at	Hk1	1.64	0.03
1425303_at	Gck	0.77	0.05
1439148_a_at	Pfkl	0.87	0.46
1419737_a_at	Ldha	1.07	0.64
1416780_at	Pfkm	0.91	0.36
1416069_at	Pfkp	1.41	0.00
1448470_at	Fbp1	1.91	0.00
1439375_x_at	Aldoa	0.82	0.01
1451194_at	Aldob	6.46	0.01
1451461_a_at	Aldoc	0.64	0.00
1415918_a_at	Tpi1	1.09	0.04
1438640_x_at	Pgk1	0.96	0.66
1426554_a_at	Pgam1	1.07	0.46
1419023_x_at	Eno1	1.23	0.05
1418829_a_at	Eno2	1.21	0.22
1417951_at	Eno3	0.79	0.01
1438711_at	Pklr	1.08	0.64
**Mitochondria**			
1418560_at	Pdha1	1.03	0.54
1448214_at	Pdhb	1.28	0.01
1426265_x_at	Dlat	1.09	0.12
1423748_at	Pdk1	1.84	0.00
1448825_at	Pdk2	1.19	0.17
1417273_at	Pdk4	1.90	0.01
1416383_a_at	Pcx	0.55	0.00
1436934_s_at	Aco2	0.85	0.04
1419821_s_at	Idh1	0.95	0.68
1416788_a_at	Idh3g	0.94	0.20
1415891_at	Suclg1	0.94	0.52
1424828_a_at	Fh1	0.95	0.67
1433984_a_at	Mdh2	1.02	0.81
1430307_a_at	Me1	1.14	0.34
1439459_x_at	Acly	0.87	0.02

**Glycerol-phosphate shuttle**			
1439396_x_at	Gpd1	0.71	0.21
1428323_at	Gpd2	0.49	0.00
**Malate-aspartate shuttle**			
1454925_x_at	Mdh1	0.60	1.03
1433984_a_at	Mdh2	0.81	1.02
1450970_at	Got1	0.58	1.16
1417715_a_at	Got2	0.16	0.88
**Electron transport chain**			
1451096_at	Ndufs2	0.65	0.00
1418117_at	Ndufs4	1.22	0.00
1448198_a_at	Ndufb8	0.82	0.03
1426688_at	Sdha	0.96	0.66
1418005_at	Sdhb	0.95	0.45
1435986_x_at	Sdhc	1.10	0.56
1428235_at	Sdhd	1.05	0.31
1428782_a_at	Uqcrc1	1.13	0.15
1428631_a_at	Uqcrc2	1.10	0.06
1417417_a_at	Cox6a1	0.95	0.50
1457633_x_at	Cox6a2	0.17	0.00
1416565_at	Cox6b1	0.98	0.83
1434491_a_at	Cox6c	0.82	0.03
1416112_at	Cox8a	0.88	0.01
1443789_x_at	Cox8c	0.55	0.00
1449710_s_at	Atp5a1	0.87	0.01
1443495_at	Atp5j2	0.70	0.03
1459740_s_at	Ucp2	1.07	0.63

Supplementary Table 2.

RT-PCR primers used for mRNA expression analysis.

Gene	Forward	Reverse
**Mouse**		
Ins1	TAGTGACCAGCTATAATCAGAG	ACGCCAAGGTCTGAAGGTCC
Ins2	CCCTGCTGGCCCTGCTCTT	AGGTCTGAAGGTCACCTGCT
Pcsk1	AGTTGGAGGCATAAGAATGCTG	GCCTTCTGGGCTAGTCTGC
Pcsk2	GTGTGATGGTTTTTGCGTCTG	GGGAGCTTTCGGACTCCAA
Abcc8	TCAACTTGTCTGGTGGTCAGC	GAGCTGAGAAAGGGTCATCCA
Kcnj11	CTGTCCCGAAAGGGCATTAT	CGTTGCAGTTGCCTTTCTTG
Pclo	TACTCGGACCCATTTGTGAA	TACTGTTTGATTCCACTCGGGATT
Rims2	CTGCTCAGCTAGTGGGACG	CCCGGATGATTTCTACCTCCAG
Sylt4	ATCATTTAGTGTGCCGAGAATGC	CCTGTTCGGTAATCAAAGCGA
Vamp2	GCTGGATGACCGTGCAGAT	GATGGCGCAGATCACTCCC
Gjd2	CAGCAGCACTCCACTATGATTG	CGTACACCGTCTCCCCTACAA
Slc30a8	CAGAGAACTTCGACAGAAGCC	CTTGCTTGCTCGACCTGTT
Glut2	TGTGATCCAGTGAGTCTCCAA	GGCGCACATCTATAATGCTCT
Gck	GATGTATTCCATCCCCGAGGACG	GCTCCACATTCTGCATCTCCTCC
Hk1	GTGGACGGGACGCTCTAC	TTCACTGTTTGGTGCATGATT
AldoB	AGAAGGACAGCCAGGGAAAT	GTTCAGAGAGGCCATCAAGC
Gapdh	CATGTTCCAGTATGACTCCACTC	GGCCTCACCCCATTTGATGT
Eno1	TGCGTCCACTGGCATCTAC	CAGAGCAGGCGCAATAGTTTTA
Ldha	AGGTTACACATCCTGGGCCATT	TCAGGAGTCAGTGTCACCTTCACA
Pcx	CTGAAGTTCCAAACAGTTCGAGG	CGCACGAAACACTCGGATG
Cox6a1	TACCCTCACCTGCGCATCAG	TCATAGCCGGTCGGAAGTGG
Atp5a1	TCTCCATGCCTCTAACACTCG	CCAGGTCAACAGACGTGTCAG
NeuroD1	GCCCAGCTTAATGCCATCTTT	CAAAAGGGCTGCCTTCTGTAA
Ngn3	GTCGGGAGAACTAGGATGGC	GGAGCAGTCCCTAGGTATG
MafA	TTCAGCAAGGAGGAGGTCAT	CTCTGGAGCTGGCACTTCTC
MafB	CTGCGCCCCTAGCCCTGGACTC	GGCGGCCCTGGCACTCACAAA
Mct1	TGTTAGTCGGAGCCTTCATTTC	CACTGGTCGTTGCACTGAATA
Ucn3	GCTGTGCCCCTCGACCT	TGGGCATCAGCATCGCT
Dlk1	CCCAGGTGAGCTTCGAGT	GGAGAGGGGTACTCTTGTTGAG
Npy	AGAGATCCAGCCCTGAGACA	GATGAGGGTGGAAACTTGGA
Oat	AGGGAAAGGGTTGCTAAATGC	CGCAGGCACACCTTCCA
Pdgfra	GACCCTGTTCCAGAGGAGGAA	TTCCGAAGTCTGTGAGCTGTGT
Igfbp4	CGGAGCTGTCGGAAATCG	TTGAAGCTGTTGTTGGGATGTT
Mylk	TGGGGGACGTGAAACTGTTTG	GGGGCAGAATGAAAGCTGG
Dnmt3a	AACGGAAACGGGATGAGTG	ACTGCAATTACCTTGGCTTTCT
Pdx1	GAAATCCACCAAAGCTCACG	CGGGTTCCGCTGTGTAAG
Nkx6.1	GAAGCGTGGTGTTGAGATGA	GGGCCCTTCCAAACAAGT
Nkx2.2	CCGGGCGGAGAAAGGTATG	CTGTAGGCGGAAAAGGGGA
Hnf4a	GAGAGGGTCAGAAGCAGACG	TTGCACAACCACAGGAAGGT
Itih5	ATCAACTGCATGTGGAGGTCA	ACTGAGACATCATTCCCCATCT
Fgf1	CCCTGACCGAGAGGTTCAAC	GTCCCTTGTCCCATCCACG

Cxcl12	CCCTGCCGGTTCTTCGA	CAGCCGTGCAACAATCTGAA
Actin	TGTACCCAGGCATTGCTGAC	CTGCTGGAAGGTGGACAGTG
**Rat**		
Dnmt3a	GCACCAGGGAAAGATCATGT	TGACGATGGAGAGGTCATTG
Actin	AGGCCCCTCTGAACCCTAAG	GGAGCGCGTAACCCTCATAG
